# Synthesis and crystal structure of a neodymium borosilicate, Nd_3_BSi_2_O_10_

**DOI:** 10.1107/S2056989019005024

**Published:** 2019-04-25

**Authors:** Saehwa Chong, Jared O. Kroll, Jarrod V. Crum, Brian J. Riley

**Affiliations:** a Pacific Northwest National Laboratory, Richland, WA 99352, USA

**Keywords:** LiCl flux, neodymium borosilicate, lanthanum borosilicate, glass-ceramic waste form, powder diffraction

## Abstract

A lanthanide borosilicate, trineodymium borosilicate or Nd_3_BSi_2_O_10_, was synthesized using a flux method with LiCl, and its structure was determined from X-ray powder diffraction (XRD) and electron probe microanalysis (EPMA). The structure is composed of layers with [SiO_4_]^4−^ and [BSiO_6_]^5−^ anions alternating along the *c* axis linked by Nd^3+^ cations between them.

## Abstract 2

The crystal structure of a lanthanide borosilicate, Nd_3_BSi_2_O_10_, has been determined from laboratory X-ray powder diffraction data. It is composed of [SiO_4_]^4−^ and [BSiO_6_]^5−^ anionic layers linked by Nd^3+^ cations between them.

## Chemical context   

Lanthanide borosilicates (*i.e.* Ln_3_BSi_2_O_10_) crystallize as one of the major phases within the residual glass matrix in some formulations of the glass-ceramic waste form for treatment of raffinate high-level waste (Crum *et al.*, 2012 ▸, 2014 ▸, 2016 ▸). Studies on the crystal chemistry and crystallization mechanism of lanthanide borosilicates are important in understanding the formation and durability of crystalline phases in the glass-ceramic waste forms (Crum *et al.*, 2012 ▸, 2014 ▸, 2016 ▸). In this work, we report the synthesis method and crystal structure of Nd_3_BSi_2_O_10_ solved by powder XRD and EPMA analysis.

Different compositions of lanthanide borosilicates including *Ln*BSiO_5_ (*Ln* = La, Ce, Pr, Nd, Sm; McAndrew & Scott, 1955 ▸; Neumann *et al.*, 1966 ▸; Nekrasov & Nekrasova, 1971 ▸; Voronkov & Pyatenko, 1967 ▸; Burns *et al.*, 1993 ▸; Chi *et al.*, 1997 ▸; Shi *et al.*, 1997 ▸), *Ln*_5_Si_2_BO_13_ (*Ln* = La, Eu, Gd, Dy; Mazza *et al.*, 2000 ▸; Yuan *et al.*, 2007 ▸; Naidu *et al.*, 2010 ▸), and *Ln*_3_BSi_2_O_10_ (*Ln* = La, Ce, Pr, Nd, Sm, Eu, Gd, Tb; Chi *et al.*, 1996*a* ▸,*b* ▸; Müller-Bunz & Schleid, 1998 ▸; Chi *et al.*, 1998 ▸, Shvanskii *et al.*, 2000 ▸; Müller-Bunz *et al.*, 2001 ▸; Bräuchle & Huppertz, 2015 ▸) have been reported in the literature. *Ln*BSiO_5_ has the stillwellite structure containing single or mixed lanthanide cations with infinite helical chains composed of six-membered rings formed by two [BO_4_]^5−^ and one [SiO_4_]^4−^ tetra­hedral units (Chi *et al.*, 1997 ▸; Burns *et al.*, 1993 ▸; Voronkov & Pyatenko, 1967 ▸; Shi *et al.*, 1997 ▸). *Ln*_5_Si_2_BO_13_ has an apatite-like structure in which the non-tetra­hedral cation sites are occupied by trivalent rare-earth cations, and B and Si occupy the same tetra­hedral site (Mazza *et al.*, 2000 ▸). *Ln*_3_BSi_2_O_10_ contains layers with [SiO_4_]^4−^ and [BSiO_6_]^5−^ anions alternating along the *c* axis linked by trivalent cations between them, and Nd_3_BSi_2_O_10_ in this work is isostructural to previously reported *Ln*_3_BSi_2_O_10_ compounds (Chi *et al.*, 1996*a* ▸,*b* ▸, 1998 ▸, Braeuchle & Huppertz, 2015 ▸; Shvanskii *et al.*, 2000 ▸; Müller–Bunz *et al.*, 2001 ▸).

## Structural commentary   

**Figure 1:**
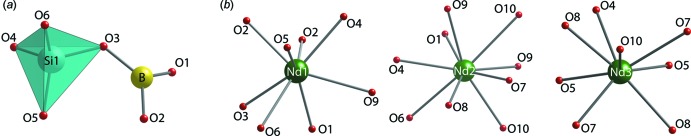
(*a*) Structure of the BSiO_6_ anion and (*b*) coordination of oxygen atoms around Nd cations (Nd1, Nd2, and Nd3).

**Figure 2:**
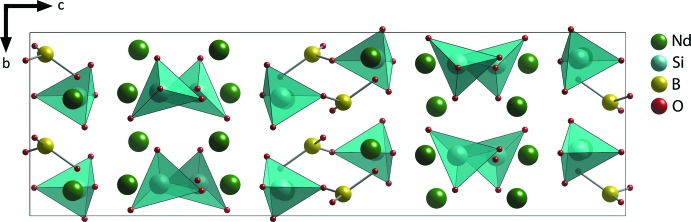
Crystal structure of Nd_3_BSi_2_O_10_ showing alternating [BSiO_6_]^5−^ and [SiO_4_]^4−^ anions along the *c* axis with Nd cations between them.

The [BSiO_6_]^5−^ anion in Nd_3_BSi_2_O_10_ is formed by [Si1O_4_]^4−^ and [BO_3_]^3−^ anions sharing an oxygen atom, with an average <Si1—O> distance of 1.613 Å and an average <B—O> distance of 1.466 Å (Fig. 1 ▸ *a*), while the [Si2O_4_]^4−^ ion has an average <Si2—O> distance of 1.590 Å. The [BSi1O_6_]^5−^ and [Si2O_4_]^4−^ anions are arranged alternately along the *c* axis (Fig. 2 ▸). The Nd cations occupy the inter­layer sites between the anion units. Nd1 and Nd3 are coordinated by eight oxygen atoms with average <Nd1—O> and <Nd3—O> distances of 2.477 and 2.520 Å, respectively, and Nd2 is coordinated by nine oxygen atoms with an average <Nd2—O> distance of 2.575 Å (Fig. 1 ▸ *b*). In our previous paper (Kroll *et al.*, 2019 ▸), we summarized the crystallographic data from the literature on other *Ln*_3_BSi_2_O_10_ chemistries (Braeuchle & Huppertz, 2015 ▸; Chi *et al.*, 1996*a* ▸,*b* ▸, 1998 ▸; Müller–Bunz *et al.*, 2001 ▸; Shvanskii *et al.*, 2000 ▸) as a function of the ionic crystal radii (*r*_c_) for the VIII-coordinated *Ln* ^3+^ constituent according to Shannon (1976 ▸) to create predictive models for the unit-cell parameters (*i.e*., *a*, *b*, and *c*), cell volume, and cell density. The measured values of *a* (9.7889 Å), *b* (7.1077 Å), *c* (23.0893 Å), cell volume (1606.5 Å^3^), and density (5.4551 Mg m^−3^) all fit reasonably well with the values calculated using the *r_c_* for Nd (1.109 Å), *i.e*., *a* (9.799 Å), *b* (7.111 Å), *c* (23.095 Å), cell volume (1608.4 Å^3^), and cell density (5.49 Mg/m^3^). Detailed atomic coordinates, bond lengths, and angles are given in Tables S1 and S2 in the supporting information.

## Synthesis and crystallization   

**Figure 3:**
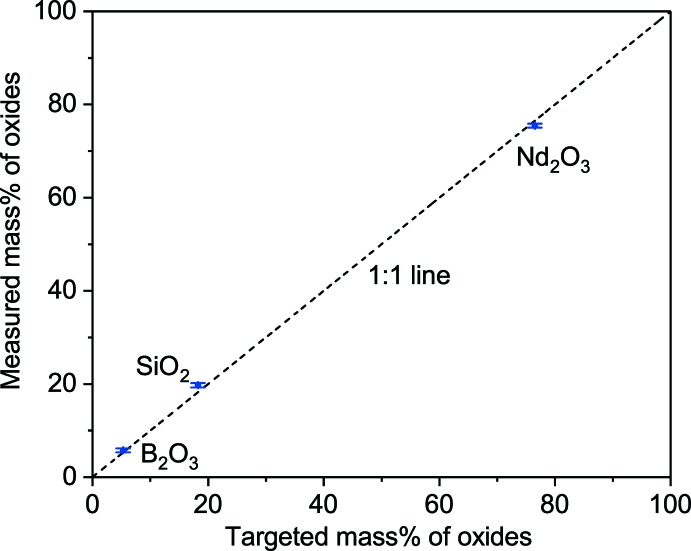
Comparison of oxide mass% between targeted and measured Nd_3_BSi_2_O_10_ from EPMA measurement.

Nd_3_BSi_2_O_10_ was synthesized by a LiCl flux method; more details are provided elsewhere (Kroll *et al.*, 2019 ▸). Powdered B_2_O_3_ was placed into a Pt–10%Rh crucible, melted at 1273 K in air to dehydrate fully and quenched on an Inconel plate. Appropriate amounts of Nd_2_O_3_, SiO_2_, and B_2_O_3_ were mixed in an agate mortar and pestle. LiCl was dried at 378 K for several hours and mixed with oxides in a 1:1 ratio by mass in a Diamonite^TM^ mortar and pestle. Mixed powder was placed into a fused quartz tube, covered with a quartz lid, heated to 1173 K at 5 K min^−1^, held for 24 h at 1173 K, and then cooled down to room temperature at 1 K min^−1^. The Nd_3_BSi_2_O_10_ was recovered from the LiCl through vacuum filtration with several rinsing steps using deionized water and a Büchner funnel. The recovered heat-treated powder was ground finer in the mortar and pestle and pressed into a 20 mm diameter pellet using a cold press with 110 MPa. The pellet was sintered at 1373 K. The heating condition included ramping up at 2 K min^−1^ to 1373 K, dwelling for 4 h, and cooling to room temperature at 2 K min^−1^. The heat-treated pellet, which was blue–violet in color, was ground for XRD and EPMA. Two EPMA measurements were performed on the sample to verify the composition of the crystal and showed that it closely matches the calculated value (Fig. 3 ▸).

## Refinement   

**Figure 4:**
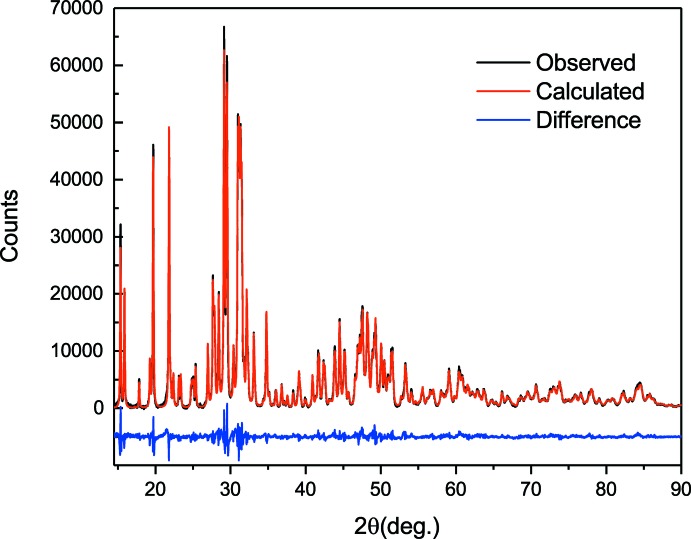
Observed, calculated, and difference XRD profiles of Nd_3_BSi_2_O_10_.

Crystal data, data collection and structure refinement details are summarized in Table 1 ▸. A Rietveld plot is shown in Fig. 4 ▸. The structure of Nd_3_BSi_2_O_10_ was determined using Rietveld refinement on the initial model with a similar chemistry and structure using *TOPAS* (version 4.2; Bruker, 2009 ▸). Based on the fitting of peak positions and profile of experimental XRD patterns to a reference pattern, Ce_3_BSi_2_O_10_ (ICSD 94423) was used as a starting model. The Ce atoms in ICSD 94423 were replaced with Nd atoms, and all the atomic positions for Nd, B, Si, and O were refined. The profile of the model was refined from 14.5° to avoid a hump around 13.5° in the fitting of the background resulting from an instrumental artifact. The displacement parameters (*B*_eq_) were not refined and fixed to 1 Å^2^ to avoid divergence and unreasonable error values. In addition, parameters for unit cell, scale factors, microstructure effects, and preferred orientation with spherical harmonic function (Järvinen, 1993 ▸) were refined, and the background was fitted with a Chebychev polynomial.

## Supplementary Material

Crystal structure: contains datablock(s) global, I. DOI: 10.1107/S2056989019005024/vn2146sup1.cif

Structure factors: contains datablock(s) I. DOI: 10.1107/S2056989019005024/vn2146Isup2.hkl

refinement details and tables for atomic positions,bond length, and angles. DOI: 10.1107/S2056989019005024/vn2146sup3.docx

Rietveld powder data: contains datablock(s) I. DOI: 10.1107/S2056989019005024/vn2146Isup4.rtv

TOPAS inp file about refinement. DOI: 10.1107/S2056989019005024/vn2146sup5.docx

CCDC reference: 1909612

Additional supporting information:  crystallographic information; 3D view; checkCIF report

## Figures and Tables

**Table 1:** Experimental details

Crystal data	
Chemical formula	Nd_3_BSi_2_O_10_
*M*_r_	659.7
Crystal system, space group	Orthorhombic, *P* *b* *c* *a*
Temperature (K)	295
*a*, *b*, *c* (Å)	9.78891 (17), 7.10774 (12), 23.0893 (4)
*V* (Å^3^)	1606.49 (5)
*Z*	8
Radiation type	Cu *K*α, λ = 1.54188 Å
Specimen shape, size (mm)	Flat sheet, 25 × 25

Data collection	
Diffractometer	Bruker D8 Advance
Specimen mounting	Packed powder pellet
Data collection mode	Reflection
Scan method	Step
2θ values (°)	2θ_min_ = 14.5 2θ_max_ = 90 2θ_step_ = 0.014

Refinement	
*R* factors and goodness of fit	*R*_p_ = 0.03, *R*_wp_ = 0.04, *R*_exp_ = 0.011, *R*_Bragg_ = 0.013, χ^2^ = 13.250
No. of parameters	82

Computer programs: *XRD Commander* (Kienle *et al.*, 2003 ▸), *TOPAS* (Bruker, 2009 ▸), *VESTA* (Momma & Izumi, 2011 ▸) and *publCIF* (Westrip, 2010 ▸).

## supplementary crystallographic information

### Crystal data

**Table:**

Nd_3_BSi_2_O_10_	*Z* = 8
*M**_r_* = 659.7	*D*_x_ = 5.455 Mg m^−^^3^
Orthorhombic, *P**b**c**a*	Cu *K*α radiation, λ = 1.54188 Å
*a* = 9.78891 (17) Å	*T* = 295 K
*b* = 7.10774 (12) Å	blue_violet
*c* = 23.0893 (4) Å	flat_sheet, 25 × 25 mm
*V* = 1606.49 (5) Å^3^	

### Data collection

**Table:**

Bruker D8 Advance diffractometer	Data collection mode: reflection
Radiation source: sealed X-ray tube	Scan method: step
Specimen mounting: packed powder pellet	2θ_min_ = 14.5°, 2θ_max_ = 90°, 2θ_step_ = 0.014°

### Refinement

**Table:**

*R*_p_ = 0.03	82 parameters
*R*_wp_ = 0.04	Weighting scheme based on measured s.u.'s
*R*_exp_ = 0.011	(Δ/σ)_max_ = 0.011
*R*_Bragg_ = 0.013	Background function: Chebychev
5738 data points	Preferred orientation correction: spherical harmonic
Profile function: pseudo-Voigt	

### Special details

**Table:**

Refinement. background fitted from 14.5 degree to avoid a hump from instrumental artifact

### Fractional atomic coordinates and isotropic or equivalent isotropic displacement parameters (Å^2^)

**Table:**

	*x*	*y*	*z*	*B*_iso_*/*B*_eq_	
Nd1	0.4909 (2)	0.3621 (3)	0.42810 (6)	1	
Nd2	0.1338 (2)	0.3296 (4)	0.33652 (7)	1	
Nd3	0.2655 (2)	0.0934 (3)	0.18257 (7)	1	
B1	0.249 (4)	0.387 (7)	0.9703 (13)	1	
Si1	0.3810 (10)	0.3516 (16)	0.0787 (3)	1	
Si2	0.4381 (9)	0.3240 (17)	0.2814 (4)	1	
O1	0.2558 (17)	0.254 (3)	0.9191 (7)	1	
O2	0.1165 (18)	0.399 (3)	0.9903 (7)	1	
O3	0.3697 (19)	0.348 (3)	0.0088 (6)	1	
O4	0.4525 (17)	0.170 (3)	0.1055 (7)	1	
O5	0.2286 (15)	0.346 (3)	0.1083 (7)	1	
O6	0.4662 (17)	0.537 (3)	0.0938 (8)	1	
O7	0.6028 (18)	0.293 (2)	0.2773 (7)	1	
O8	0.4151 (15)	0.369 (2)	0.2120 (7)	1	
O9	0.3903 (18)	0.466 (2)	0.3239 (7)	1	
O10	0.3481 (15)	0.138 (3)	0.2880 (6)	1	

### Atomic displacement parameters (Å^2^)

**Table:**

	*U*^11^	*U*^22^	*U*^33^	*U*^12^	*U*^13^	*U*^23^
?	?	?	?	?	?	?

### Geometric parameters (Å, º)

**Table:**

Nd1—O1^i^	2.454 (17)	Nd3—O7^vi^	2.325 (16)
Nd1—O2^ii^	2.460 (19)	B1—O2	1.38 (4)
Nd1—O2^iii^	2.265 (17)	Si1—O3	1.618 (16)
Nd1—O4^iv^	2.39 (2)	Si1—O4	1.59 (2)
Nd1—O6^v^	2.40 (2)	Si1—O5	1.641 (18)
Nd2—O1^i^	2.327 (17)	Si1—O6	1.60 (2)
Nd2—O8^vi^	2.432 (15)	Si2—O7	1.63 (2)
Nd2—O10^vii^	2.47 (2)	Si2—O8	1.649 (19)
Nd3—Nd3^viii^	3.567 (3)	Si2—O9	1.483 (19)
Nd3—Nd3^vii^	3.567 (3)	Si2—O10	1.60 (2)
Nd3—O5^viii^	2.457 (19)		
			
O1^i^—Nd1—O2^ii^	83.2 (6)	O3—Si1—O6	105.4 (11)
O1^i^—Nd1—O2^iii^	128.2 (6)	O4—Si1—O5	102.6 (11)
O1^i^—Nd1—O4^iv^	119.9 (6)	O4—Si1—O6	110.7 (11)
O1^i^—Nd1—O6^v^	79.8 (6)	O5—Si1—O6	113.8 (11)
O2^ii^—Nd1—O2^iii^	70.5 (6)	O7—Si2—O8	96.0 (9)
O2^ii^—Nd1—O4^iv^	69.9 (6)	O7—Si2—O9	116.3 (11)
O2^ii^—Nd1—O6^v^	149.5 (6)	O7—Si2—O10	116.1 (11)
O2^iii^—Nd1—O4^iv^	92.2 (7)	O8—Si2—O9	117.9 (11)
O2^iii^—Nd1—O6^v^	101.0 (7)	O8—Si2—O10	100.3 (9)
O4^iv^—Nd1—O6^v^	140.6 (6)	O9—Si2—O10	109.0 (10)
O1^i^—Nd2—O8^vi^	149.1 (6)	Nd1^ix^—O1—Nd2^ix^	117.7 (7)
O1^i^—Nd2—O10^vii^	124.2 (6)	Nd1^x^—O2—Nd1^xi^	109.5 (7)
O8^vi^—Nd2—O10^vii^	75.6 (5)	Nd1^x^—O2—B1	104 (2)
Nd3^viii^—Nd3—Nd3^vii^	170.24 (8)	Nd1^xi^—O2—B1	141.4 (17)
Nd3^viii^—Nd3—O5^viii^	44.7 (4)	Nd1^v^—O4—Si1	135.9 (10)
Nd3^viii^—Nd3—O7^vi^	123.3 (4)	Nd3^vii^—O5—Si1	104.6 (9)
Nd3^vii^—Nd3—O5^viii^	135.7 (4)	Nd1^iv^—O6—Si1	147.0 (11)
Nd3^vii^—Nd3—O7^vi^	48.2 (4)	Nd3^xii^—O7—Si2	137.5 (9)
O5^viii^—Nd3—O7^vi^	137.0 (5)	Nd2^xii^—O8—Si2	107.8 (8)
O3—Si1—O4	113.9 (11)	Nd2^viii^—O10—Si2	137.7 (9)
O3—Si1—O5	110.7 (10)		

Symmetry codes: (i) *x*, −*y*+1/2, *z*−1/2; (ii) −*x*+1/2, −*y*+1, *z*−1/2; (iii) *x*+1/2, *y*, −*z*+3/2; (iv) −*x*+1, *y*+1/2, −*z*+1/2; (v) −*x*+1, *y*−1/2, −*z*+1/2; (vi) *x*−1/2, *y*, −*z*+1/2; (vii) −*x*+1/2, *y*+1/2, *z*; (viii) −*x*+1/2, *y*−1/2, *z*; (ix) *x*, −*y*+1/2, *z*+1/2; (x) −*x*+1/2, −*y*+1, *z*+1/2; (xi) *x*−1/2, *y*, −*z*+3/2; (xii) *x*+1/2, *y*, −*z*+1/2.

## References

[bb2] Bräuchle, S. & Huppertz, H. (2015). *Z. Naturforsch. Teil B*, **70**, 929–934.

[bb3] Bruker (2009). *TOPAS*., Bruker AXS, Karlsruhe, Germany.

[bb4] Burns, P. C., Hawthorne, F. C., MacDonald, D. J., della Ventura, G. & Parodi, G. C. (1993). *Can. Mineral.* **31**, 147–152.

[bb5] Chi, L., Chen, H., Deng, S., Zhuang, H. & Huang, J. (1996*a*). *Acta Cryst.* C**52**, 2385–2387.

[bb6] Chi, L., Chen, H., Deng, S., Zhuang, H. & Huang, J. (1996*b*). *J. Alloys Compd.* **242**, 1–5.

[bb7] Chi, L., Chen, H., Lin, X., Zhuang, H. & Huang, J. (1998). *J. Struct. Chem. China*, **17**, 297–301.

[bb8] Chi, L., Chen, H., Zhuang, H. & Huang, J. (1997). *J. Alloys Compd.* **252**, L12–L15.

[bb9] Crum, J., Maio, V., McCloy, J., Scott, C., Riley, B., Benefiel, B., Vienna, J., Archibald, K., Rodriguez, C., Rutledge, V., Zhu, Z., Ryan, J. & Olszta, M. (2014). *J. Nucl. Mater.* **444**, 481–492.

[bb10] Crum, J. V., Neeway, J. J., Riley, B. J., Zhu, Z., Olszta, M. J. & Tang, M. (2016). *J. Nucl. Mater.* **482**, 1–11.

[bb11] Crum, J. V., Turo, L., Riley, B., Tang, M. & Kossoy, A. (2012). *J. Am. Ceram. Soc.* **95**, 1297–1303.

[bb12] Järvinen, M. (1993). *J. Appl. Cryst.* **26**, 525–531.

[bb13] Kienle, M. & Jacob, M. (2003). *DIFFRAC* plus *XRD Commander*. Bruker AXS GmbH, Karlsruhe, Germany.

[bb14] Kroll, J. O., Crum, J. V., Riley, B. J., Neeway, J. J., Asmussen, R. M. & Liezers, M. (2019). *J. Nucl. Mater.* **515**, 370–381.

[bb15] Mazza, D., Tribaudino, M., Delmastro, A. & Lebech, B. (2000). *J. Solid State Chem.* **155**, 389–393.

[bb16] McAndrew, J. & Scott, T. (1955). *Nature*, **176**, 509–510.

[bb17] Momma, K. & Izumi, F. (2011). *J. Appl. Cryst.* **44**, 1272–1276.

[bb18] Müller-Bunz, H., Grossholz, H. & Schleid, T. (2001). *Z. Anorg. Allg. Chem.* **627**, 1436–1438.

[bb19] Müller-Bunz, H. & Schleid, T. (1998). *Z. Kristallogr. Suppl.* **15**, 48.

[bb1] Naidu, S. A., Varadaraju, U. V. & Raveau, B. (2010). *J. Solid State Chem.* **183**, 1847–1852.

[bb20] Nekrasov, I. I. & Nekrasova, R. A. (1971). *Dokl. Akad. Nauk SSSR*, **201**, 1202.

[bb21] Neumann, H., Bergstøl, S. & Nilssen, B. (1966). *Nor. Geo. Tidsskr*, **46**, 327–334.

[bb22] Shannon, R. D. (1976). *Acta Cryst.* A**32**, 751–767.

[bb23] Shi, Y., Liang, J. K., Zhang, H., Yang, J. L., Zhuang, W. D. & Rao, G. H. (1997). *J. Alloys Compd.* **259**, 163–169.

[bb24] Shvanskii, E. V., Leonyuk, N. I., Bocelli, G. & Righi, L. (2000). *J. Solid State Chem.* **154**, 312–316.

[bb25] Voronkov, A. A. & Pyatenko, Y. A. (1967). *Sov. Phys. Cryst.* **12**, 258–265.

[bb26] Westrip, S. P. (2010). *J. Appl. Cryst.* **43**, 920–925.

[bb27] Yuan, J.-L., Zhang, Z.-J., Wang, X.-J., Chen, H.-H., Zhao, J.-T., Zhang, G.-B. & Shi, C.-S. (2007). *J. Solid State Chem.* **180**, 1365–1371.

