# Supplementary material for: Synthesis and crystal structure of a neodymium borosilicate, Nd3BSi2O10
Source: Acta Crystallogr E Crystallogr Commun. 2019 Apr 25;75(Pt 5):700–2. doi: 10.1107/S2056989019005024 (PMC6505600; doi:10.1107/S2056989019005024)
Supplement: Supplementary file 3 [file e-75-00700-sup3.docx]

# Supporting information

***Synthesis and crystal structure of a neodymium borosilicate Nd_3_BSi_2_O_10_***

Saehwa Chong, Jared O. Kroll, Jarrod V. Crum, Brian J. Riley

Computing details

Data collection: *XRD commander* (Kienle & Jacob, 2003); program used to solve and refine structure: *TOPAS* (Bruker, 2009); molecular graphics: *VESTA* (Momma & Izumi, 2011); software used to prepare materials for publication: *publ*CIF (Westrip, 2010).

*Crystal data*

| Nd_3_BSi_2_O_10_ | *V* = 1606.47(5) Å^3^ |
| --- | --- |
| *M_r_* = 659.7 | *Z* = 8 |
| Orthorhombic, *Pbca* | *D_x_* = 5.4547 Mg m^-3^ |
| Hall symbol: -P 2ac 2ab | Cu *K*α_1_, *K*α_2_, λ = 1.540562, 1.544390 Å |
| *a* = 9.7889(2) Å | *T* = 295 K |
| *b* = 7.10770(10) Å | blue-violet |
| *c* = 23.0893(4) Å | cylinder, 25 × 1 mm |

*Data collection*

| Bruker AXS D8 Advance diffractometer | Data collection mode: reflection |
| --- | --- |
| Radiation source: sealed X-ray tube, BrukerAXS D8 | scan method: step |
| Specimen mounting: standard PMMA Ø 25 mm back loading holder | 2θ_min_ = 14.5, 2θ_max_ = 90, 2θ_step_ = 0.014 |

*Refinement*

| *R*_p_ = 0.030 | *R*_Bragg_ = 0.013 |
| --- | --- |
| *R*_wp_ = 0.040 | 82 parameters |
| *R*_exp_ = 0.011 | Background function: Chebyshev polynomial |
|  |  |

Table S1. Atomic coordinates. Displacement parameters (B_eq_) were fixed as 1 Å^2^ during refinement.

| Atom | *x* | y | z |
| --- | --- | --- | --- |
| Nd1 | 0.4909(2) | 0.3621(3) | 0.42810(6) |
| Nd2 | 0.1338(2) | 0.3296(4) | 0.33652(7) |
| Nd3 | 0.2655(2) | 0.0934(3) | 0.18257(7) |
| B1 | 0.249(4) | 0.387(7) | 0.9703(13) |
| Si1 | 0.3810(10) | 0.3516(16) | 0.0787(3) |
| Si2 | 0.4381(9) | 0.3240(17) | 0.2814(4) |
| O1 | 0.2558(17) | 0.254(3) | 0.9191(7) |
| O2 | 0.1165(18) | 0.399(3) | 0.9903(7) |
| O3 | 0.3697(19) | 0.348(3) | 0.0088(6) |
| O4 | 0.4525(17) | 0.170(3) | 0.1055(7) |
| O5 | 0.2286(15) | 0.346(3) | 0.1083(7) |
| O6 | 0.4662(17) | 0.537(3) | 0.0938(8) |
| O7 | 0.6028(18) | 0.293(2) | 0.2773(7) |
| O8 | 0.4151(15) | 0.369(2) | 0.2120(7) |
| O9 | 0.3903(18) | 0.466(2) | 0.3239(7) |
| O10 | 0.3481(15) | 0.138(3) | 0.2880(6) |

Table S2. Bond lengths and angles.

| Geometric parameters (Å, º) | | | | |
| --- | --- | --- | --- | --- |
| Nd1-O1^i^ | 2.454(17) |  | O1^i^-Nd1-O2^ii^ | 83.2(6) |
| Nd1-O2^ii^ | 2.460(19) |  | O1^i^-Nd1-O2^iii^ | 128.2(6) |
| Nd1-O2^iii^ | 2.265(17) |  | O1^i^-Nd1-O4^iv^ | 119.9(6) |
| Nd1-O4^iv^ | 2.39(2) |  | O1^i^-Nd1-O6^v^ | 79.8(6) |
| Nd1-O5^v^ | 2.40(2) |  | O2^ii^-Nd1-O2^iii^ | 70.5(6) |
| Nd2-O1^i^ | 2.327(17) |  | O2^i^i-Nd1-O4^iv^ | 69.9(6) |
| Nd2-O8^vi^ | 2.432(15) |  | O2^ii^-Nd1-O6 | 149.5(6) |
| Nd2-O10^vii^ | 2.47(2) |  | O2^iii^-Nd1-O4^iv^ | 92.2(7) |
| Nd3-Nd3^vii^ | 3.567(3) |  | O2^iii^-Nd1-O6^v^ | 101.0(7) |
| Nd3-Nd3^viii^ | 3.567(3) |  | O4^iv^-Nd1-O6^v^ | 140.6(6) |
| Nd3-O5 | 2.457(19) |  | O1^i^-Nd2-O8^vi^ | 149.1(6) |
| Nd3-O7^vi^ | 2.325(16) |  | O1^i^-Nd2-O10^vii^ | 124.2(6) |
| B1-O2 | 1.38(4) |  | O8^vi^-Nd2-O10^vii^ | 75.6(5) |
| Si1-O3 | 1.618(16) |  | Nd3^viii^-Nd3-Nd3^vii^ | 170.24(8) |
| Si1-O4 | 1.59(2) |  | Nd3^viii^-Nd3-O5^viii^ | 44.7(4) |
| Si1-O5 | 1.641(18) |  | Nd3^viii^-Nd3-O7^vi^ | 123.3(4) |
| Si1-O6 | 1.60(2) |  | Nd3^vii^-Nd3-O5^viii^ | 135.7(4) |
| Si2-O7 | 1.63(2) |  | Nd3^vii^-Nd3-O7^vi^ | 48.2(4) |
| Si2-O8 | 1.649(19) |  | O5^viii^-Nd3-O7^vi^ | 137.0(5) |
| Si2-O9 | 1.483(19) |  | O3-Si1-O4 | 113.9(11) |
| Si2-O10 | 1.60(2) |  | O3-Si1-O5 | 110.7(10) |
|  |  |  | O3-Si1-O6 | 105.4(11) |
|  |  |  | O4-Si1-O5 | 102.6(11) |
|  |  |  | O4-Si1-O6 | 110.7(11) |
|  |  |  | O5-Si1-O6 | 113.8(11) |
|  |  |  | O7-Si2-O8 | 96.0(9) |
|  |  |  | O7-Si2-O9 | 116.3(11) |
|  |  |  | O7-Si2-O10 | 116.1(11) |
|  |  |  | O8-Si2-O9 | 117.9(11) |
|  |  |  | O8-Si2-O10 | 100.3(9) |
|  |  |  | O9-Si2-O10 | 109.0(10) |
|  |  |  | Nd1i^x^-O1-Nd2^ix^ | 117.7(7) |
|  |  |  | Nd1^x^-O2-Nd1^xi^ | 109.5(7) |
|  |  |  | Nd1^x^-O2-B1 | 104(2) |
|  |  |  | Nd1^xi^-O2-B1 | 141.4(17) |
|  |  |  | Nd1^v^-O4-Si1 | 135.9(10) |
|  |  |  | Nd3^vii^-O5-Si1 | 104.6(9) |
|  |  |  | Nd1^iv^-O6-Si1 | 147.0(11) |
|  |  |  | Nd3^xii^-O7-Si2 | 137.5(9) |
|  |  |  | Nd2^xii^-O8-Si2 | 107.8(8) |
|  |  |  | Nd2^viii^-O10-Si2 | 137.7(9) |

Symmetry codes: (i) x,-y+1/2,z-1/2; (ii) -x+1/2,-y+1,z-1/2; (iii) x+1/2,y,-z+3/2; (iv) -x+1,y+1/2,-z+1/2; (v) x+1/2,y,-z+1/2; (vi) x-1/2,y,-z+1/2; (vii) -x+1/2,y-1/2,z; (viii) -x+1/2,y+1/2,z; (ix) x,-y+1/2,z+1/2; (x) -x+1/2,-y+1,z+1/2; (xi) x-1/2,y,-z+3/2; (xii) x+1/2,y,-z+1/2

Bruker, A. X. S. (2009). TOPAS, Version 4.2., Bruker AXS, Karlsruhe, Germany.

Kienle, M. & Jacob, M. (2003). DIFFRAC plus XRD Commander.

Momma, K. & Izumi, F. (2011). *J. Appl. Crys.* **44**, 1272-1276.

Westrip, S. P. (2010). *J. Appl. Crys.* **43**, 920-925.
