# Supplementary material for: Synthesis and crystal structure of a neodymium borosilicate, Nd3BSi2O10
Source: Acta Crystallogr E Crystallogr Commun. 2019 Apr 25;75(Pt 5):700–2. doi: 10.1107/S2056989019005024 (PMC6505600; doi:10.1107/S2056989019005024)
Supplement: Supplementary file 5 [file e-75-00700-sup5.docx]

r_exp 1.10413067 r_exp_dash 2.05325172 r_wp 4.02234355 r_wp_dash 7.47998769 r_p 2.99632578 r_p_dash 6.66327217 weighted_Durbin_Watson 0.254001017 gof 3.64299595

iters 100000

do_errors

prm lsmax =Get(refine_ls_shift_on_su_max); : 0.01090

xdd "C:\Users\chon120\OneDrive - PNNL\Desktop\saehwa pnnl\Crum project\___Crystal data\Data for Nd3BSi2O10\Nd3BSi2O10 1100°C_4hr (24sec per step) 3-22-19.raw"

r_exp 1.10413067 r_exp_dash 2.05325172 r_wp 4.02234355 r_wp_dash 7.47998769 r_p 2.99632578 r_p_dash 6.66327217 weighted_Durbin_Watson 0.254001017 gof 3.64299595

range 1

bkg @ 6506.03164`_9.40571721 -8651.68826`_14.9918235 5757.0693`_14.4265293 -3440.32013`_13.5820319 1913.50267`_12.7873866 -1085.79899`_11.9161094 478.725213`_11.5552002 -247.269941`_11.0404998 145.972115`_10.2844346 -2.1602732`_9.88884418 -43.8174584`_9.97832611 133.457297`_8.71291616 -21.3246113`_7.06449139

start_X 14.5

LP_Factor( 0)

Specimen_Displacement(@, -0.02762`_0.00098)

Rp 250

Rs 250

lpsd_th2_angular_range_degrees 3

lpsd_equitorial_divergence_degrees 0.3

lpsd_equitorial_sample_length_mm 10

lpsd_beam_spill_correct_intensity 0

axial_conv

filament_length 12

sample_length 25

receiving_slit_length 12

primary_soller_angle 2.5

secondary_soller_angle 2.5

axial_n_beta 30

lam

ymin_on_ymax 0.0001

la 0.653817 lo 1.540596 lh 0.501844

la 0.346183 lo 1.544493 lh 0.626579

str

LVol_FWHM_CS_G_L( 1, 106.38141`_2.45528, 0.89, 148.72214`_3.43251,,,@, 167.10353`_3.85675 min =50;)

e0_from_Strain( 0.00037`_0.00001,,,@, 0.16980`_0.00434 max =1;)

r_bragg 1.3352435

phase_MAC 272.645308

phase_name "Nd3BSi2O10"

MVW( 5277.568, 1606.486`_0.047, 100.000`_0.000)

scale @ 0.000178291495`_4.254e-007

space_group Pbca

Phase_LAC_1_on_cm( 1487.31960`_0.04348)

Phase_Density_g_on_cm3( 5.45514`_0.00016)

a @ 9.788915`_0.000170

b @ 7.107742`_0.000120

c @ 23.089299`_0.000375

site Nd1 num_posns 8 x @ 0.49085`_0.00021 y @ 0.36208`_0.00032 z @ 0.42810`_0.00006 occ Nd+3 1 beq 1

site Nd2 num_posns 8 x @ 0.13384`_0.00021 y @ 0.32962`_0.00036 z @ 0.33652`_0.00007 occ Nd+3 1 beq 1

site Nd3 num_posns 8 x @ 0.26551`_0.00020 y @ 0.09341`_0.00030 z @ 0.18257`_0.00007 occ Nd+3 1 beq 1

site B1 num_posns 8 x @ 0.24950`_0.00378 y @ 0.38712`_0.00702 z @ 0.97030`_0.00128 occ B 1 beq 1

site Si1 num_posns 8 x @ 0.38097`_0.00097 y @ 0.35158`_0.00162 z @ 0.07871`_0.00032 occ Si+4 1 beq 1

site Si2 num_posns 8 x @ 0.43806`_0.00086 y @ 0.32405`_0.00169 z @ 0.28138`_0.00039 occ Si+4 1 beq 1

site O1 num_posns 8 x @ 0.25576`_0.00171 y @ 0.25378`_0.00259 z @ 0.91911`_0.00075 occ O-2 1 beq 1

site O2 num_posns 8 x @ 0.11655`_0.00180 y @ 0.39939`_0.00251 z @ 0.99027`_0.00069 occ O-2 1 beq 1

site O3 num_posns 8 x @ 0.36967`_0.00187 y @ 0.34760`_0.00291 z @ 0.00879`_0.00057 occ O-2 1 beq 1

site O4 num_posns 8 x @ 0.45246`_0.00165 y @ 0.16979`_0.00280 z @ 0.10546`_0.00066 occ O-2 1 beq 1

site O5 num_posns 8 x @ 0.22854`_0.00151 y @ 0.34581`_0.00263 z @ 0.10827`_0.00070 occ O-2 1 beq 1

site O6 num_posns 8 x @ 0.46620`_0.00173 y @ 0.53735`_0.00259 z @ 0.09383`_0.00075 occ O-2 1 beq 1

site O7 num_posns 8 x @ 0.60278`_0.00181 y @ 0.29329`_0.00244 z @ 0.27733`_0.00067 occ O-2 1 beq 1

site O8 num_posns 8 x @ 0.41508`_0.00149 y @ 0.36912`_0.00249 z @ 0.21205`_0.00066 occ O-2 1 beq 1

site O9 num_posns 8 x @ 0.39031`_0.00180 y @ 0.46623`_0.00247 z @ 0.32392`_0.00072 occ O-2 1 beq 1

site O10 num_posns 8 x @ 0.34813`_0.00152 y @ 0.13793`_0.00272 z @ 0.28796`_0.00063 occ O-2 1 beq 1

PO_Spherical_Harmonics(sh_595a92bc_10, 8 load sh_Cij_prm { y00 !sh_595a92bc_10_c00 1 y20 sh_595a92bc_10_c20 0.15478`_0.00651 y22p sh_595a92bc_10_c22p 0.07472`_0.00392 y40 sh_595a92bc_10_c40 0.10248`_0.00846 y42p sh_595a92bc_10_c42p -0.03434`_0.00646 y44p sh_595a92bc_10_c44p 0.04591`_0.00723 y60 sh_595a92bc_10_c60 0.04368`_0.00947 y62p sh_595a92bc_10_c62p 0.00805`_0.00638 y64p sh_595a92bc_10_c64p 0.07503`_0.00526 y66p sh_595a92bc_10_c66p 0.02704`_0.00587 y80 sh_595a92bc_10_c80 0.11531`_0.00940 y82p sh_595a92bc_10_c82p -0.00594`_0.00848 y84p sh_595a92bc_10_c84p 0.06158`_0.00534 y86p sh_595a92bc_10_c86p -0.05437`_0.00678 y88p sh_595a92bc_10_c88p 0.01725`_0.00811 } )

C_matrix_normalized

{

1 2 3 4 5 6 7 8 9 10 11 12 13 14 15 16 17 18 19 20 21 22 23 24 25 26 27 28 29 30 31 32 33 34 35 36 37 38 39 40 41 42 43 44 45 46 47 48 49 50 51 52 53 54 55 56 57 58 59 60 61 62 63 64 65 66 67 68 69 70 71 72 73 74 75 76 77 78 79 80 81 82

bkgb717da8 1: 100 -75 60 -51 54 -48 40 -37 37 -20 9 6 6 2 -0 2 1 -2 -5 2 0 5 10 3 -3 -7 5 3 11 -4 -51 -4 -1 -2 0 -6 2 2 6 -0 -0 4 5 -0 4 8 -5 11 3 3 1 3 2 -4 4 2 -0 -4 4 7 6 -6 3 -7 2 4 1 -1 7 -9 -9 -1 3 3 -0 2 -5 -1 -13 -2 -5 -3

bkgb717e48 2: -75 100 -79 80 -66 63 -50 49 -37 34 -20 18 -4 -1 2 -3 3 4 1 6 3 -2 4 0 1 -1 0 -3 -19 -13 9 1 0 0 -0 2 -2 -1 -2 2 3 -1 1 -3 -2 -5 -0 -4 5 3 0 -1 4 -3 -10 -5 3 1 -6 -3 -1 1 -3 1 -5 2 6 2 1 5 4 3 0 -1 -5 -3 3 -1 6 2 -1 3

bkgb717ee8 3: 60 -79 100 -79 80 -61 63 -46 45 -34 40 -27 20 -1 -1 0 -4 -4 6 -7 -4 -5 -9 -5 2 6 -11 -3 17 20 22 2 2 -0 6 -3 2 -7 -1 -1 -4 -2 7 5 3 3 3 -1 -3 -8 -2 2 -1 9 7 6 -2 3 -1 5 1 6 2 -5 -2 -3 -3 -0 -0 0 3 -2 -3 0 7 1 -1 -1 -4 -3 2 0

bkgb717f88 4: -51 80 -79 100 -81 77 -60 56 -43 45 -33 35 -13 -0 2 -4 5 -0 1 8 -1 1 6 -2 3 6 -9 -2 -7 -10 -7 0 -1 1 5 -2 -1 1 -3 2 4 2 3 -8 3 -8 -1 -1 8 1 2 1 9 -1 -13 -1 2 5 -11 6 -0 -3 -7 -4 -6 3 10 5 8 4 5 -2 -2 0 -4 -2 4 -1 1 -5 0 3

bkgb718028 5: 54 -66 80 -81 100 -77 74 -54 56 -43 42 -28 19 1 2 4 -4 -1 1 -1 5 -5 -5 -4 1 -6 4 0 2 5 5 0 0 -2 1 -3 1 -5 2 -1 -2 -1 5 7 3 7 -1 0 -3 -0 -4 -4 -3 1 12 -1 -0 -0 5 0 4 5 3 -4 1 -2 -0 -4 -3 -1 -4 4 2 3 5 1 -3 -4 -4 3 0 0

bkgb7180c8 6: -48 63 -61 77 -77 100 -74 73 -55 49 -30 24 -14 0 -2 -1 -0 -0 1 1 -1 -1 2 5 1 8 -7 5 1 1 7 1 0 -0 6 -2 -2 5 -3 -2 5 -4 -5 0 1 -4 -3 -2 3 -6 2 5 2 3 -11 5 -1 -3 -2 4 1 -4 -3 1 -2 -2 -3 -0 1 11 0 -4 -4 -4 3 7 6 1 6 -3 10 -1

bkgb718168 7: 40 -50 63 -60 74 -74 100 -71 70 -48 41 -28 23 0 0 2 3 -0 0 -6 -0 -6 -4 -6 6 5 4 -7 3 5 7 1 -1 1 6 3 5 -11 -4 -1 3 1 4 2 6 4 6 1 -8 -1 -1 -1 0 -1 11 -3 -0 4 -1 1 -3 1 3 -4 -4 1 3 6 -6 3 3 -2 -1 -0 4 -7 -10 -0 3 -2 1 -6

bkgb718208 8: -37 49 -46 56 -54 73 -71 100 -71 62 -39 34 -18 0 -1 1 -0 1 9 1 6 -4 -7 4 3 -6 -10 -1 -4 -3 5 1 -0 -1 11 -1 -6 -1 -2 0 -1 -3 2 6 1 2 -7 -0 -9 9 5 9 4 5 -8 0 -0 4 -6 -1 2 -3 -4 3 -7 -0 2 3 6 3 5 -0 3 4 -2 14 -2 -5 8 -0 12 7

bkgb7182a8 9: 37 -37 45 -43 56 -55 70 -71 100 -70 59 -34 33 1 5 -1 -3 -3 1 -0 2 3 3 -5 -3 -1 4 -2 1 -1 -6 -1 -1 -2 1 3 1 -6 3 -3 1 -1 2 -9 2 -5 5 -4 7 -0 5 -6 -6 1 6 -7 -2 -4 -3 1 -5 3 5 0 2 -1 -1 -2 -1 -1 -0 -0 5 -5 3 -2 1 7 -9 2 -1 -2

bkgb718348 10: -20 34 -34 45 -43 49 -48 62 -70 100 -65 59 -34 1 -7 -3 -0 4 -1 1 -6 -0 -0 2 0 0 -10 -2 -0 -3 -3 -1 -0 -1 -0 -11 4 1 4 -2 -6 -5 -1 5 -4 5 1 1 1 1 -5 -3 4 1 -9 5 -3 7 2 -6 2 -4 -3 2 -8 2 -1 3 9 -7 4 2 -3 4 1 -4 5 -7 7 -8 -7 10

bkgb7183e8 11: 9 -20 40 -33 42 -30 41 -39 59 -65 100 -65 47 -0 9 2 2 -0 5 -2 4 0 2 -7 6 6 -8 -0 4 12 25 2 2 -3 -2 1 5 -2 -2 2 -5 2 2 0 8 -12 7 -4 2 -10 -8 -8 3 -2 8 -0 6 1 -6 7 7 4 -11 3 11 -5 2 -5 -4 4 1 -3 -4 2 -1 3 11 -13 -4 -1 8 -7

bkgb718488 12: 6 18 -27 35 -28 24 -28 34 -34 59 -65 100 -41 1 -2 -5 -0 -6 0 11 2 -4 6 2 2 -4 -11 -4 -3 -14 -26 -1 -0 -3 11 1 3 -2 2 -1 3 -6 12 -7 -6 -0 -6 5 5 14 -5 0 10 5 -20 7 -4 2 -3 4 5 -0 10 -3 -13 -4 15 5 16 -5 12 5 -3 1 1 4 -1 -1 -15 -6 -11 11

bkgb718528 13: 6 -4 20 -13 19 -14 23 -18 33 -34 47 -41 100 -2 -6 -8 -1 -10 -5 -2 0 -2 4 -5 -4 6 6 -2 -2 2 8 2 1 1 7 18 2 -9 -12 10 10 2 3 9 -6 6 -8 -1 8 0 12 7 1 -5 -4 6 4 -8 -8 0 7 5 9 -15 -13 1 3 5 -5 -7 5 1 10 -8 -6 -8 -13 8 -4 11 4 -1

m5ca22fd5_188 14: 2 -1 -1 -0 1 0 0 0 1 1 -0 1 -2 100 0 3 -1 -1 -1 -1 -1 6 -0 1 -3 -2 5 2 -4 -5 -5 -69 -67 -71 -0 -4 -0 6 2 -1 -3 2 1 -1 -1 1 3 0 -3 -2 4 -3 -2 0 4 0 -4 2 -0 -5 -1 -1 2 1 5 3 -0 1 5 1 -0 2 4 2 4 -1 2 4 4 2 4 -5

sh_595a92bc_10_c20 15: -0 2 -1 2 2 -2 0 -1 5 -7 9 -2 -6 0 100 -6 13 18 -1 26 4 -6 -9 -41 19 -6 4 -2 2 3 1 6 -3 -1 -10 0 13 -1 9 14 -2 19 8 2 -4 -6 -0 -16 -5 -4 7 -4 -8 4 6 -11 -11 23 -4 1 -3 9 -16 -15 -4 6 4 -1 -21 20 2 4 -24 11 -7 7 23 -0 -19 -7 -3 -7

sh_595a92bc_10_c22p 16: 2 -3 0 -4 4 -1 2 1 -1 -3 2 -5 -8 3 -6 100 7 -1 8 -13 13 -23 -1 2 -22 -8 8 2 -0 1 -3 -1 -3 4 10 -6 -2 10 -3 -9 -0 8 -3 -6 6 -8 -2 11 -21 6 -5 11 5 -2 6 2 12 6 -3 -6 -8 -9 6 8 3 3 -8 -12 -6 9 -7 6 7 10 -0 7 -5 -16 21 1 7 -18

sh_595a92bc_10_c40 17: 1 3 -4 5 -4 -0 3 -0 -3 -0 2 -0 -1 -1 13 7 100 18 -13 -3 -5 -8 17 -12 13 5 21 -11 0 1 -4 -5 6 6 -2 15 18 -6 14 -3 11 18 -1 -3 -5 -1 2 25 -1 -3 -7 -17 18 -12 9 18 13 25 -4 3 4 6 10 7 -3 18 10 2 0 3 5 -30 -2 -2 -14 12 -15 5 -7 -1 -6 -29

sh_595a92bc_10_c42p 18: -2 4 -4 -0 -1 -0 -0 1 -3 4 -0 -6 -10 -1 18 -1 18 100 -9 12 -24 -10 -3 -1 17 -12 11 -19 -3 -1 -7 -0 8 3 -11 -2 3 3 20 11 -1 7 -12 10 -0 21 0 6 5 -15 -6 3 -3 8 25 -9 -1 6 12 -6 2 9 -11 27 14 16 -42 24 -9 22 3 -17 -8 6 0 5 20 1 3 -20 -12 -5

sh_595a92bc_10_c44p 19: -5 1 6 1 1 1 0 9 1 -1 5 0 -5 -1 -1 8 -13 -9 100 -26 6 1 -20 10 3 -19 -48 -32 -2 0 16 3 1 2 16 3 -2 -20 1 -3 -20 9 11 -7 4 -7 14 -16 -3 -5 -1 -5 -4 42 -3 -3 15 1 -30 -16 -14 -20 -26 9 -7 -7 -2 -4 6 6 4 23 10 13 -1 -13 -4 -6 -0 -19 1 11

sh_595a92bc_10_c60 20: 2 6 -7 8 -1 1 -6 1 -0 1 -2 11 -2 -1 26 -13 -3 12 -26 100 -15 -8 -18 -19 -7 4 -0 20 -7 -9 -10 7 -0 -7 -3 -14 -7 -4 -12 14 11 15 19 -1 -3 27 -14 -1 30 13 -13 -11 9 19 2 0 -3 3 -12 17 4 28 1 -9 -14 1 26 -3 4 8 4 18 -23 -14 10 21 20 2 -32 -17 -16 -2

sh_595a92bc_10_c62p 21: 0 3 -4 -1 5 -1 -0 6 2 -6 4 2 0 -1 4 13 -5 -24 6 -15 100 -2 12 -7 21 -14 1 29 -6 -6 -7 3 -5 0 13 -5 -16 -2 -3 -12 1 -14 -3 11 -0 -14 -3 -8 -18 21 -2 -0 -5 -14 -10 -5 -10 -7 12 -8 -8 -14 -9 -6 0 -6 6 -22 4 18 1 17 9 0 -5 5 -0 -13 -12 8 11 5

sh_595a92bc_10_c64p 22: 5 -2 -5 1 -5 -1 -6 -4 3 -0 0 -4 -2 6 -6 -23 -8 -10 1 -8 -2 100 -6 30 5 -5 -7 3 -1 -4 -13 -9 -6 -1 -29 -14 5 13 15 -9 -15 -7 -5 -3 -13 -10 6 -7 -5 -8 11 -7 5 -12 1 -6 -25 1 2 -14 -11 4 -10 24 17 9 -7 -7 27 -2 -5 -1 9 9 2 -12 -3 11 -5 9 -2 -15

sh_595a92bc_10_c66p 23: 10 4 -9 6 -5 2 -4 -7 3 -0 2 6 4 -0 -9 -1 17 -3 -20 -18 12 -6 100 12 -1 28 15 9 -11 -13 -24 -6 4 -2 -7 2 -2 10 6 -12 15 -12 1 22 -8 -9 -15 19 14 1 -19 -5 -1 -39 -10 9 1 -10 11 -0 23 -16 15 -1 7 14 -4 -5 6 -1 -7 -7 9 -20 -26 -15 4 -12 -17 4 -14 -8

sh_595a92bc_10_c80 24: 3 0 -5 -2 -4 5 -6 4 -5 2 -7 2 -5 1 -41 2 -12 -1 10 -19 -7 30 12 100 -12 -7 -5 -1 -3 -6 -15 -11 3 -4 -8 4 -2 7 9 4 -1 -11 -7 6 -14 14 4 10 -11 4 6 6 -9 -13 3 -9 -14 -20 18 -13 8 -16 -8 34 12 -3 -16 11 9 -0 1 -18 -1 -6 13 9 -15 -9 0 13 3 -17

sh_595a92bc_10_c82p 25: -3 1 2 3 1 1 6 3 -3 0 6 2 -4 -3 19 -22 13 17 3 -7 21 5 -1 -12 100 2 -9 -31 3 2 9 5 3 5 11 19 -6 -0 11 8 4 -1 -8 -1 -0 -2 2 0 -12 -5 -10 -4 19 -17 8 15 -12 10 -6 8 8 7 -16 2 -6 7 -2 12 -7 21 11 -27 -9 17 -9 6 6 -8 -12 -10 15 7

sh_595a92bc_10_c84p 26: -7 -1 6 6 -6 8 5 -6 -1 0 6 -4 6 -2 -6 -8 5 -12 -19 4 -14 -5 28 -7 2 100 6 6 10 12 26 1 4 4 12 -4 -0 -15 -15 10 12 -5 10 5 3 1 -11 0 6 -19 -3 -1 12 -2 -10 -6 -2 24 6 19 7 -1 8 -2 -1 3 -1 11 -12 3 1 -21 -1 -19 -11 -19 12 9 -15 -9 6 -10

sh_595a92bc_10_c86p 27: 5 0 -11 -9 4 -7 4 -10 4 -10 -8 -11 6 5 4 8 21 11 -48 -0 1 -7 15 -5 -9 6 100 15 -12 -13 -35 -8 -4 2 -22 17 0 2 -8 5 23 4 -12 11 6 19 -14 11 -5 10 4 -5 -17 -32 5 -7 10 -6 26 1 6 -6 27 1 11 5 -16 3 -32 1 -29 -16 21 -10 -3 2 -8 19 10 19 4 -25

sh_595a92bc_10_c88p 28: 3 -3 -3 -2 0 5 -7 -1 -2 -2 -0 -4 -2 2 -2 2 -11 -19 -32 20 29 3 9 -1 -31 6 15 100 0 -0 -9 1 -7 -6 1 -12 -0 33 1 1 9 -8 -7 20 -6 7 -20 11 -1 11 3 9 -16 8 -12 12 -1 -12 3 7 -5 -20 3 -7 8 -11 -13 -21 17 9 -0 -5 11 -18 16 9 18 8 -15 2 11 -16

m5ca22fd5_186 29: 11 -19 17 -7 2 1 3 -4 1 -0 4 -3 -2 -4 2 -0 0 -3 -2 -7 -6 -1 -11 -3 3 10 -12 0 100 92 7 4 4 3 4 -4 3 0 7 -3 -11 3 -0 -5 -0 -1 2 2 -5 -10 1 7 -3 6 7 8 -6 4 5 5 -0 7 -3 -3 2 -1 -4 -1 -3 0 -0 -4 -5 6 9 3 3 5 -0 -1 2 -2

m5ca22fd5_187 30: -4 -13 20 -10 5 1 5 -3 -1 -3 12 -14 2 -5 3 1 1 -1 0 -9 -6 -4 -13 -6 2 12 -13 -0 92 100 27 7 4 4 3 -4 1 0 7 -4 -13 5 1 -3 -2 -3 3 -1 -7 -14 0 8 -2 7 7 9 -5 6 4 2 -2 11 -4 -4 3 1 -4 -1 -4 3 -0 -3 -5 5 9 1 4 5 4 -2 4 -5

scalecaf130_ 31: -51 9 22 -7 5 7 7 5 -6 -3 25 -26 8 -5 1 -3 -4 -7 16 -10 -7 -13 -24 -15 9 26 -35 -9 7 27 100 9 4 3 13 1 2 -10 -8 -0 -6 -7 4 1 0 -12 11 -16 -7 -15 -3 -0 4 21 -1 6 -3 15 -11 0 -7 13 -7 1 -8 -9 2 2 -5 11 19 -2 -12 -2 6 -3 6 -2 10 -6 10 6

a118d30b8_ 32: -4 1 2 0 0 1 1 1 -1 -1 2 -1 2 -69 6 -1 -5 -0 3 7 3 -9 -6 -11 5 1 -8 1 4 7 9 100 39 38 9 -0 -1 -7 -7 1 4 0 -1 3 4 -0 -5 -3 4 5 -2 3 3 4 -6 -1 3 -1 -4 7 -4 4 -4 -10 -8 -8 5 -5 -8 6 3 4 -9 -1 -1 3 1 -5 -2 2 -0 5

b118d3138_ 33: -1 0 2 -1 0 0 -1 -0 -1 -0 2 -0 1 -67 -3 -3 6 8 1 -0 -5 -6 4 3 3 4 -4 -7 4 4 4 39 100 33 -1 2 3 -11 -3 4 2 -7 -6 -2 2 2 -2 1 4 -3 -9 -0 6 3 -0 1 6 -3 5 5 2 4 -4 5 -5 -3 -0 -5 -8 -4 0 -3 -5 -2 -2 3 -2 -7 -5 -1 -8 5

c118d31b8_ 34: -2 0 -0 1 -2 -0 1 -1 -2 -1 -3 -3 1 -71 -1 4 6 3 2 -7 0 -1 -2 -4 5 4 2 -6 3 4 3 38 33 100 -2 4 -3 -3 -1 1 3 9 2 0 2 -2 -1 4 0 4 2 6 -4 -1 -2 1 4 4 -2 1 -7 -3 1 0 -2 3 -3 2 -5 2 -3 -5 1 -4 -7 1 -6 4 4 -1 -5 0

xb7185c8_ 35: 0 -0 6 5 1 6 6 11 1 -0 -2 11 7 -0 -10 10 -2 -11 16 -3 13 -29 -7 -8 11 12 -22 1 4 3 13 9 -1 -2 100 12 -13 -4 -15 8 6 -4 1 -6 2 9 -29 13 -11 10 -2 5 23 13 -4 9 12 -3 -15 14 -6 -2 11 -17 -6 -11 8 17 7 16 30 1 17 8 11 7 -5 -7 -4 -14 17 0

yb718668_ 36: -6 2 -3 -2 -3 -2 3 -1 3 -11 1 1 18 -4 0 -6 15 -2 3 -14 -5 -14 2 4 19 -4 17 -12 -4 -4 1 -0 2 4 12 100 5 -7 6 8 7 -2 -10 8 -5 4 1 26 -1 12 -3 -6 -2 -5 2 20 17 -10 1 -1 8 -10 18 13 6 -12 -5 18 -7 -15 11 -12 8 0 -23 -5 -17 7 -3 2 8 -5

zb718708_ 37: 2 -2 2 -1 1 -2 5 -6 1 4 5 3 2 -0 13 -2 18 3 -2 -7 -16 5 -2 -2 -6 -0 0 -0 3 1 2 -1 3 -3 -13 5 100 5 -3 3 8 10 -4 2 8 -4 14 -1 3 -13 8 -10 2 0 -11 8 1 -2 -4 6 23 -9 -15 8 6 -15 1 3 -17 -4 -1 -6 -10 3 -2 -11 -2 0 12 15 4 -10

xb7187a8_ 38: 2 -1 -7 1 -5 5 -11 -1 -6 1 -2 -2 -9 6 -1 10 -6 3 -20 -4 -2 13 10 7 -0 -15 2 33 0 0 -10 -7 -11 -3 -4 -7 5 100 15 6 -23 3 -24 -6 -12 -13 -16 14 -9 -6 -0 14 14 -14 6 20 3 -6 13 -1 1 -12 -0 -1 14 -4 -14 -7 14 6 4 -6 -13 8 -1 -5 36 3 4 14 12 -15

yb718848_ 39: 6 -2 -1 -3 2 -3 -4 -2 3 4 -2 2 -12 2 9 -3 14 20 1 -12 -3 15 6 9 11 -15 -8 1 7 7 -8 -7 -3 -1 -15 6 -3 15 100 -11 -21 -15 -6 2 -35 -8 11 2 -7 -11 12 11 -9 -6 5 15 -15 -0 2 -36 -5 1 -5 24 22 19 -19 -5 18 -15 5 -14 5 1 -8 -2 13 40 -5 -7 15 -7

zb7188e8_ 40: -0 2 -1 2 -1 -2 -1 0 -3 -2 2 -1 10 -1 14 -9 -3 11 -3 14 -12 -9 -12 4 8 10 5 1 -3 -4 -0 1 4 1 8 8 3 6 -11 100 9 0 -14 -3 5 14 -14 -4 -15 -5 8 -2 3 19 22 -11 -7 9 4 2 6 -3 -8 -5 -1 -4 -5 5 -16 -6 20 -11 -12 16 -8 -8 2 -4 -19 4 -6 -1

xb718988_ 41: -0 3 -4 4 -2 5 3 -1 1 -6 -5 3 10 -3 -2 -0 11 -1 -20 11 1 -15 15 -1 4 12 23 9 -11 -13 -6 4 2 3 6 7 8 -23 -21 9 100 1 4 15 6 7 -11 -1 9 10 -6 -4 10 -14 -3 -7 -3 2 1 23 17 -1 9 -20 -7 -1 16 8 -15 15 -11 -23 -9 -15 8 10 -20 -12 -1 3 6 -1

yb718a28_ 42: 4 -1 -2 2 -1 -4 1 -3 -1 -5 2 -6 2 2 19 8 18 7 9 15 -14 -7 -12 -11 -1 -5 4 -8 3 5 -7 0 -7 9 -4 -2 10 3 -15 0 1 100 14 -14 12 4 7 3 23 1 -11 -5 6 -1 4 -2 18 9 -18 14 2 9 -5 -4 -2 8 6 8 -8 -7 -6 9 2 -0 5 2 10 3 -6 -6 -9 -4

zb718ac8_ 43: 5 1 7 3 5 -5 4 2 2 -1 2 12 3 1 8 -3 -1 -12 11 19 -3 -5 1 -7 -8 10 -12 -7 -0 1 4 -1 -6 2 1 -10 -4 -24 -6 -14 4 14 100 -2 2 1 7 -3 25 14 8 9 1 4 -22 -2 -3 23 -29 10 1 16 3 -12 -20 2 28 11 20 1 -12 12 3 -13 3 4 -8 -0 -9 -9 -6 13

xb718b68_ 44: -0 -3 5 -8 7 0 2 6 -9 5 0 -7 9 -1 2 -6 -3 10 -7 -1 11 -3 22 6 -1 5 11 20 -5 -3 1 3 -2 0 -6 8 2 -6 2 -3 15 -14 -2 100 -19 17 -6 3 3 6 3 1 -24 3 -6 21 -9 -12 14 -24 30 -18 -12 -4 -5 4 -21 11 -6 18 -3 -6 7 -24 -5 0 -10 -1 -4 -3 2 -8

yb718c08_ 45: 4 -2 3 3 3 1 6 1 2 -4 8 -6 -6 -1 -4 6 -5 -0 4 -3 -0 -13 -8 -14 -0 3 6 -6 -0 -2 0 4 2 2 2 -5 8 -12 -35 5 6 12 2 -19 100 0 9 1 4 3 -14 -6 8 3 1 -18 8 11 -8 25 -9 -4 6 2 -2 -11 6 -2 -11 2 -6 1 5 12 -9 9 -0 -23 18 1 8 5

zb718ca8_ 46: 8 -5 3 -8 7 -4 4 2 -5 5 -12 -0 6 1 -6 -8 -1 21 -7 27 -14 -10 -9 14 -2 1 19 7 -1 -3 -12 -0 2 -2 9 4 -4 -13 -8 14 7 4 1 17 0 100 -13 9 10 2 3 2 -7 29 17 -11 4 -23 19 0 16 -2 4 5 -10 7 -15 8 -8 9 -6 2 11 -6 1 -2 3 12 -6 -5 -1 -2

xb718d48_ 47: -5 -0 3 -1 -1 -3 6 -7 5 1 7 -6 -8 3 -0 -2 2 0 14 -14 -3 6 -15 4 2 -11 -14 -20 2 3 11 -5 -2 -1 -29 1 14 -16 11 -14 -11 7 7 -6 9 -13 100 -13 1 -10 8 8 -13 -3 -1 -14 -11 -2 -13 -8 -11 7 -16 23 17 -4 -13 4 1 -7 -16 5 -10 1 2 -14 -4 12 15 11 -3 -11

yb718de8_ 48: 11 -4 -1 -1 0 -2 1 -0 -4 1 -4 5 -1 0 -16 11 25 6 -16 -1 -8 -7 19 10 0 0 11 11 2 -1 -16 -3 1 4 13 26 -1 14 2 -4 -1 3 -3 3 1 9 -13 100 -5 10 -11 1 1 -2 -0 16 8 -6 -7 -7 2 -24 18 -4 -1 1 -6 20 18 -1 9 -12 20 -4 -13 15 -7 -4 5 -2 -3 -11

zb718e88_ 49: 3 5 -3 8 -3 3 -8 -9 7 1 2 5 8 -3 -5 -21 -1 5 -3 30 -18 -5 14 -11 -12 6 -5 -1 -5 -7 -7 4 4 0 -11 -1 3 -9 -7 -15 9 23 25 3 4 10 1 -5 100 8 -18 -19 -1 -4 -13 4 8 -8 -7 10 22 10 -0 0 -5 -11 11 4 7 -22 -9 10 -4 -20 3 -4 14 -1 -23 -5 -21 16

xb718f28_ 50: 3 3 -8 1 -0 -6 -1 9 -0 1 -10 14 0 -2 -4 6 -3 -15 -5 13 21 -8 1 4 -5 -19 10 11 -10 -14 -15 5 -3 4 10 12 -13 -6 -11 -5 10 1 14 6 3 2 -10 10 8 100 4 -8 -1 -13 -12 -4 5 -3 -3 -0 -3 -9 8 -0 -13 -13 14 2 7 -7 21 6 3 -19 -8 21 -17 -20 -11 15 14 12

yb718fc8_ 51: 1 0 -2 2 -4 2 -1 5 5 -5 -8 -5 12 4 7 -5 -7 -6 -1 -13 -2 11 -19 6 -10 -3 4 3 1 0 -3 -2 -9 2 -2 -3 8 -0 12 8 -6 -11 8 3 -14 3 8 -11 -18 4 100 19 -17 -4 -7 -17 -23 5 0 -11 -11 -2 -4 -3 -1 4 -10 12 -1 -5 -2 -17 7 -8 5 -7 -19 31 19 33 25 -11

zb719068_ 52: 3 -1 2 1 -4 5 -1 9 -6 -3 -8 0 7 -3 -4 11 -17 3 -5 -11 -0 -7 -5 6 -4 -1 -5 9 7 8 -0 3 -0 6 5 -6 -10 14 11 -2 -4 -5 9 1 -6 2 8 1 -19 -8 19 100 -0 -3 -1 4 -6 -8 -10 0 -14 4 9 -2 -4 2 -14 0 11 9 -3 -4 -8 3 4 21 -20 28 18 3 8 -16

xb719108_ 53: 2 4 -1 9 -3 2 0 4 -6 4 3 10 1 -2 -8 5 18 -3 -4 9 -5 5 -1 -9 19 12 -17 -16 -3 -2 4 3 6 -4 23 -2 2 14 -9 3 10 6 1 -24 8 -7 -13 1 -1 -1 -17 -0 100 -8 -1 6 12 8 -0 25 5 16 7 -16 -8 10 25 -10 16 6 4 -3 -16 10 -10 -8 10 -22 -14 2 8 3

yb7191a8_ 54: -4 -3 9 -1 1 3 -1 5 1 1 -2 5 -5 0 4 -2 -12 8 42 19 -14 -12 -39 -13 -17 -2 -32 8 6 7 21 4 3 -1 13 -5 0 -14 -6 19 -14 -1 4 3 3 29 -3 -2 -4 -13 -4 -3 -8 100 10 7 11 5 -20 -6 -9 -3 -14 0 -15 -1 -8 -9 3 10 16 17 12 -3 4 -2 7 8 -5 -27 -8 7

zb719248_ 55: 4 -10 7 -13 12 -11 11 -8 6 -9 8 -20 -4 4 6 6 9 25 -3 2 -10 1 -10 3 8 -10 5 -12 7 7 -1 -6 -0 -2 -4 2 -11 6 5 22 -3 4 -22 -6 1 17 -1 -0 -13 -12 -7 -1 -1 10 100 -7 -6 2 17 -3 8 22 -2 16 3 18 -6 -11 -5 13 10 -3 -3 17 -0 -5 -8 -8 18 -10 -9 -4

xb7192e8_ 56: 2 -5 6 -1 -1 5 -3 0 -7 5 -0 7 6 0 -11 2 18 -9 -3 0 -5 -6 9 -9 15 -6 -7 12 8 9 6 -1 1 1 9 20 8 20 15 -11 -7 -2 -2 21 -18 -11 -14 16 4 -4 -17 4 6 7 -7 100 21 -4 1 -9 15 2 -4 -5 8 -0 7 -12 14 2 22 -9 7 -18 1 3 -3 8 -3 -20 4 -17

yb719388_ 57: -0 3 -2 2 -0 -1 -0 -0 -2 -3 6 -4 4 -4 -11 12 13 -1 15 -3 -10 -25 1 -14 -12 -2 10 -1 -6 -5 -3 3 6 4 12 17 1 3 -15 -7 -3 18 -3 -9 8 4 -11 8 8 5 -23 -6 12 11 -6 21 100 -9 -2 35 3 -13 16 -3 6 -8 4 -12 5 -4 -1 7 14 -4 -11 0 4 -8 4 -10 -4 -5

zb719428_ 58: -4 1 3 5 -0 -3 4 4 -4 7 1 2 -8 2 23 6 25 6 1 3 -7 1 -10 -20 10 24 -6 -12 4 6 15 -1 -3 4 -3 -10 -2 -6 -0 9 2 9 23 -12 11 -23 -2 -6 -8 -3 5 -8 8 5 2 -4 -9 100 -12 -0 -10 8 -8 -6 -7 -6 12 21 5 6 1 -17 -2 -3 1 6 1 -11 -3 -8 5 6

xb7194c8_ 59: 4 -6 -1 -11 5 -2 -1 -6 -3 2 -6 -3 -8 -0 -4 -3 -4 12 -30 -12 12 2 11 18 -6 6 26 3 5 4 -11 -4 5 -2 -15 1 -4 13 2 4 1 -18 -29 14 -8 19 -13 -7 -7 -3 0 -10 -0 -20 17 1 -2 -12 100 -3 10 2 -9 9 21 2 -9 -8 -20 2 -24 -10 -14 -1 -10 -6 10 -10 2 21 -2 7

yb719568_ 60: 7 -3 5 6 0 4 1 -1 1 -6 7 4 0 -5 1 -6 3 -6 -16 17 -8 -14 -0 -13 8 19 1 7 5 2 0 7 5 1 14 -1 6 -1 -36 2 23 14 10 -24 25 0 -8 -7 10 -0 -11 0 25 -6 -3 -9 35 -0 -3 100 6 16 11 -19 -12 -6 17 -1 1 18 -4 -7 -11 3 1 3 5 -18 -5 -2 3 3

zb719608_ 61: 6 -1 1 -0 4 1 -3 2 -5 2 7 5 7 -1 -3 -8 4 2 -14 4 -8 -11 23 8 8 7 6 -5 -0 -2 -7 -4 2 -7 -6 8 23 1 -5 6 17 2 1 30 -9 16 -11 2 22 -3 -11 -14 5 -9 8 15 3 -10 10 6 100 3 -4 5 -0 3 8 9 -2 -1 10 -8 -8 -2 -3 -1 3 -13 -7 -3 -2 2

xb7196a8_ 62: -6 1 6 -3 5 -4 1 -3 3 -4 4 -0 5 -1 9 -9 6 9 -20 28 -14 4 -16 -16 7 -1 -6 -20 7 11 13 4 4 -3 -2 -10 -9 -12 1 -3 -1 9 16 -18 -4 -2 7 -24 10 -9 -2 4 16 -3 22 2 -13 8 2 16 3 100 10 -5 -1 14 13 -18 6 5 -8 17 -12 6 12 -1 -9 13 -9 -10 -16 -0

yb719748_ 63: 3 -3 2 -7 3 -3 3 -4 5 -3 -11 10 9 2 -16 6 10 -11 -26 1 -9 -10 15 -8 -16 8 27 3 -3 -4 -7 -4 -4 1 11 18 -15 -0 -5 -8 9 -5 3 -12 6 4 -16 18 -0 8 -4 9 7 -14 -2 -4 16 -8 -9 11 -4 10 100 -7 -15 13 9 -5 29 -7 -1 8 17 -4 -3 11 -12 19 8 4 -2 -6

zb7197e8_ 64: -7 1 -5 -4 -4 1 -4 3 0 2 3 -3 -15 1 -15 8 7 27 9 -9 -6 24 -1 34 2 -2 1 -7 -3 -4 1 -10 5 0 -17 13 8 -1 24 -5 -20 -4 -12 -4 2 5 23 -4 0 -0 -3 -2 -16 0 16 -5 -3 -6 9 -19 5 -5 -7 100 31 -12 -30 4 0 -14 6 -9 12 -5 -1 11 1 1 -2 -8 -6 -12

xb719888_ 65: 2 -5 -2 -6 1 -2 -4 -7 2 -8 11 -13 -13 5 -4 3 -3 14 -7 -14 0 17 7 12 -6 -1 11 8 2 3 -8 -8 -5 -2 -6 6 6 14 22 -1 -7 -2 -20 -5 -2 -10 17 -1 -5 -13 -1 -4 -8 -15 3 8 6 -7 21 -12 -0 -1 -15 31 100 -8 -19 -3 2 -8 -1 -6 5 12 1 -9 10 6 6 -1 5 -22

yb719928_ 66: 4 2 -3 3 -2 -2 1 -0 -1 2 -5 -4 1 3 6 3 18 16 -7 1 -6 9 14 -3 7 3 5 -11 -1 1 -9 -8 -3 3 -11 -12 -15 -4 19 -4 -1 8 2 4 -11 7 -4 1 -11 -13 4 2 10 -1 18 -0 -8 -6 2 -6 3 14 13 -12 -8 100 -3 -7 25 9 0 -1 6 12 -20 -1 -4 12 2 -12 -5 -1

zb7199c8_ 67: 1 6 -3 10 -0 -3 3 2 -1 -1 2 15 3 -0 4 -8 10 -42 -2 26 6 -7 -4 -16 -2 -1 -16 -13 -4 -4 2 5 -0 -3 8 -5 1 -14 -19 -5 16 6 28 -21 6 -15 -13 -6 11 14 -10 -14 25 -8 -6 7 4 12 -9 17 8 13 9 -30 -19 -3 100 -17 4 -15 1 13 -24 1 -5 1 -8 -17 -11 13 -2 10

xb719a68_ 68: -1 2 -0 5 -4 -0 6 3 -2 3 -5 5 5 1 -1 -12 2 24 -4 -3 -22 -7 -5 11 12 11 3 -21 -1 -1 2 -5 -5 2 17 18 3 -7 -5 5 8 8 11 11 -2 8 4 20 4 2 12 0 -10 -9 -11 -12 -12 21 -8 -1 9 -18 -5 4 -3 -7 -17 100 -1 7 7 -21 5 2 13 10 4 -0 9 -7 12 1

yb719b08_ 69: 7 1 -0 8 -3 1 -6 6 -1 9 -4 16 -5 5 -21 -6 0 -9 6 4 4 27 6 9 -7 -12 -32 17 -3 -4 -5 -8 -8 -5 7 -7 -17 14 18 -16 -15 -8 20 -6 -11 -8 1 18 7 7 -1 11 16 3 -5 14 5 5 -20 1 -2 6 29 0 2 25 4 -1 100 1 15 4 18 8 7 13 1 -1 -6 -17 4 5

zb719ba8_ 70: -9 5 0 4 -1 11 3 3 -1 -7 4 -5 -7 1 20 9 3 22 6 8 18 -2 -1 -0 21 3 1 9 0 3 11 6 -4 2 16 -15 -4 6 -15 -6 15 -7 1 18 2 9 -7 -1 -22 -7 -5 9 6 10 13 2 -4 6 2 18 -1 5 -7 -14 -8 9 -15 7 1 100 2 -4 -6 -2 15 12 5 -8 -5 -17 11 -18

xb719c48_ 71: -9 4 3 5 -4 0 3 5 -0 4 1 12 5 -0 2 -7 5 3 4 4 1 -5 -7 1 11 1 -29 -0 -0 -0 19 3 0 -3 30 11 -1 4 5 20 -11 -6 -12 -3 -6 -6 -16 9 -9 21 -2 -3 4 16 10 22 -1 1 -24 -4 10 -8 -1 6 -1 0 1 7 15 2 100 -6 5 13 -0 17 3 -9 -12 -6 11 2

yb719ce8_ 72: -1 3 -2 -2 4 -4 -2 -0 -0 2 -3 5 1 2 4 6 -30 -17 23 18 17 -1 -7 -18 -27 -21 -16 -5 -4 -3 -2 4 -3 -5 1 -12 -6 -6 -14 -11 -23 9 12 -6 1 2 5 -12 10 6 -17 -4 -3 17 -3 -9 7 -17 -10 -7 -8 17 8 -9 -6 -1 13 -21 4 -4 -6 100 4 8 4 -15 -2 -2 -3 -1 -16 12

zb719d88_ 73: 3 0 -3 -2 2 -4 -1 3 5 -3 -4 -3 10 4 -24 7 -2 -8 10 -23 9 9 9 -1 -9 -1 21 11 -5 -5 -12 -9 -5 1 17 8 -10 -13 5 -12 -9 2 3 7 5 11 -10 20 -4 3 7 -8 -16 12 -3 7 14 -2 -14 -11 -8 -12 17 12 5 6 -24 5 18 -6 5 4 100 -0 -6 -1 -17 12 3 -11 0 -5

xb719e28_ 74: 3 -1 0 0 3 -4 -0 4 -5 4 2 1 -8 2 11 10 -2 6 13 -14 0 9 -20 -6 17 -19 -10 -18 6 5 -2 -1 -2 -4 8 0 3 8 1 16 -15 -0 -13 -24 12 -6 1 -4 -20 -19 -8 3 10 -3 17 -18 -4 -3 -1 3 -2 6 -4 -5 12 12 1 2 8 -2 13 8 -0 100 -2 -1 0 -11 14 -9 7 5

yb719ec8_ 75: -0 -5 7 -4 5 3 4 -2 3 1 -1 1 -6 4 -7 -0 -14 0 -1 10 -5 2 -26 13 -9 -11 -3 16 9 9 6 -1 -2 -7 11 -23 -2 -1 -8 -8 8 5 3 -5 -9 1 2 -13 3 -8 5 4 -10 4 -0 1 -11 1 -10 1 -3 12 -3 -1 1 -20 -5 13 7 15 -0 4 -6 -2 100 17 1 0 8 -12 -2 -11

zb719f68_ 76: 2 -3 1 -2 1 7 -7 14 -2 -4 3 4 -8 -1 7 7 12 5 -13 21 5 -12 -15 9 6 -19 2 9 3 1 -3 3 3 1 7 -5 -11 -5 -2 -8 10 2 4 0 9 -2 -14 15 -4 21 -7 21 -8 -2 -5 3 0 6 -6 3 -1 -1 11 11 -9 -1 1 10 13 12 17 -15 -1 -1 17 100 -9 -8 9 -15 4 10

xb71a008_ 77: -5 3 -1 4 -3 6 -10 -2 1 5 11 -1 -13 2 23 -5 -15 20 -4 20 -0 -3 4 -15 6 12 -8 18 3 4 6 1 -2 -6 -5 -17 -2 36 13 2 -20 10 -8 -10 -0 3 -4 -7 14 -17 -19 -20 10 7 -8 -3 4 1 10 5 3 -9 -12 1 10 -4 -8 4 1 5 3 -2 -17 0 1 -9 100 -3 -18 -13 6 7

yb71a0a8_ 78: -1 -1 -1 -1 -4 1 -0 -5 7 -7 -13 -1 8 4 -0 -16 5 1 -6 2 -13 11 -12 -9 -8 9 19 8 5 5 -2 -5 -7 4 -7 7 0 3 40 -4 -12 3 -0 -1 -23 12 12 -4 -1 -20 31 28 -22 8 -8 8 -8 -11 -10 -18 -13 13 19 1 6 12 -17 -0 -1 -8 -9 -2 12 -11 0 -8 -3 100 2 4 15 -21

zb71a148_ 79: -13 6 -4 1 -4 6 3 8 -9 7 -4 -15 -4 4 -19 21 -7 3 -0 -32 -12 -5 -17 0 -12 -15 10 -15 -0 4 10 -2 -5 4 -4 -3 12 4 -5 -19 -1 -6 -9 -4 18 -6 15 5 -23 -11 19 18 -14 -5 18 -3 4 -3 2 -5 -7 -9 8 -2 6 2 -11 9 -6 -5 -12 -3 3 14 8 9 -18 2 100 8 18 -2

xb71a1e8_ 80: -2 2 -3 -5 3 -3 -2 -0 2 -8 -1 -6 11 2 -7 1 -1 -20 -19 -17 8 9 4 13 -10 -9 19 2 -1 -2 -6 2 -1 -1 -14 2 15 14 -7 4 3 -6 -9 -3 1 -5 11 -2 -5 15 33 3 2 -27 -10 -20 -10 -8 21 -2 -3 -10 4 -8 -1 -12 13 -7 -17 -17 -6 -1 -11 -9 -12 -15 -13 4 8 100 8 -5

yb71a288_ 81: -5 -1 2 0 0 10 1 12 -1 -7 8 -11 4 4 -3 7 -6 -12 1 -16 11 -2 -14 3 15 6 4 11 2 4 10 -0 -8 -5 17 8 4 12 15 -6 6 -9 -6 2 8 -1 -3 -3 -21 14 25 8 8 -8 -9 4 -4 5 -2 3 -2 -16 -2 -6 5 -5 -2 12 4 11 11 -16 0 7 -2 4 6 15 18 8 100 -9

zb71a328_ 82: -3 3 0 3 0 -1 -6 7 -2 10 -7 11 -1 -5 -7 -18 -29 -5 11 -2 5 -15 -8 -17 7 -10 -25 -16 -2 -5 6 5 5 0 0 -5 -10 -15 -7 -1 -1 -4 13 -8 5 -2 -11 -11 16 12 -11 -16 3 7 -4 -17 -5 6 7 3 2 -0 -6 -12 -22 -1 10 1 5 -18 2 12 -5 5 -11 10 7 -21 -2 -5 -9 100

}
